# ***Segue:*** Brief summaries of articles on pertinent emerging issues published elsewhere.

**DOI:** 10.3201/eid1007.040412

**Published:** 2004-07

**Authors:** Joseph E. McDade

**Affiliations:** Founding Editor of Emerging Infectious Diseases, Centers for Disease Control and Prevention, Atlanta, Georgia, USA

**Keywords:** conference summary, open access publishing

## West Nile Virus

### Epidemic of West Nile Virus in the United States, 2002

In 2002, West Nile virus (WNV) dramatically expanded its geographic range in the United States and caused the largest recognized epidemic of neuroinvasive arboviral diseases in the Western Hemisphere. That year, an estimated 180,000–1,200,000 cases of WNV infections occurred in humans in the United States. The Centers for Disease Control and Prevention's ArboNet surveillance system received reports from 39 state health departments, 29 of which reported cases for the first time, and Washington, D.C., of 4,146 WNV cases. Clinical and demographic information indicated that 2,942 (71%) were neuroinvasive (meningitis or encephalitis), 1,157 (28%) were West Nile fever, and 47 (1%) were clinically nonspecific. The fatality-to-case ratio was 9% among patients with neuroinvasive illness and 12% among patients with encephalitis. Neuroinvasive illness and death were strongly associated with advancing age and occurred slightly more frequently among males than females. Neuroinvasive illnesses occurred from mid-May to mid-December with peak incidence during the week of August 24; 84% occurred in 11 midwestern states in the Mississippi and Ohio River basins. Observations during 1999–2002 suggest that, in coming years, WNV will assume an epidemiologic pattern of intense seasonal transmission in the United States, with hundreds to thousands of human cases reported annually. WNV detection in nonhuman species appears to be a sensitive but relatively nonspecific predictor of impending transmission to humans. WNV prevention should emphasize organized, well-funded, sustained mosquito-control programs that stress the reduction of *Culex* mosquitoes, and public education messages that stress personal protection from mosquito exposure and elimination of periresidential mosquito habitats.

### West Nile Virus: An Overview of its Spread in Europe and the Mediterranean Basin in Contrast to its Spread in the Americas

West Nile infection was considered a minor arbovirosis in the Old World despite several outbreaks with encephalitis cases in the 1950s in Israel. From 1994 to 2003, West Nile outbreaks were reported in humans and horses in Algeria, Romania, Russia, Israel, Tunisia, Morocco, and France with neuroinvasive forms and fatalities mainly in elderly persons. Vectors are mosquitoes principally from the *Culex* genus, but few isolates of West Nile virus have been obtained. Birds are amplifying hosts for the virus and are considered resistant to the disease. However, the occurrence of an abnormal number of deaths in some bird species in Israel in 1998 indicated that a more virulent strain had emerged, which surprisingly reached New York City in 1999 and spread in the New World. Phylogenetic studies have shown two lineages of West Nile strains in sub-Saharan Africa, but only strains from lineage 1 were identified in the Mediterranean region and southern Europe. European authorities are concerned about new modes of transmission through blood donations and organ transplants, which occurred in the United States in 2002. An enhanced surveillance for West Nile infection in humans, horses, birds, and vectors may indicate that the virus is present in different locations, but the occurrence of outbreaks is still unpredictable.

### Emerging Vectors in the *Culex pipiens* Complex

These authors compared New World and Old World populations of *Culex pipiens* due to this taxon's likely importance as a vector of West Nile virus (WNV): on its abundance, high rates of WNV infection, peak biting activity, positive vector competence, and transovarial virus transmission. By using polymorphisms at eight nuclear microsatellite loci, they first demonstrated that American and North European populations of *Cx. pipiens* are genetically different. Also, by using multilocus fingerprinting techniques, they demonstrated that a large proportion of *Cx. pipiens* in the northeastern United States are hybrids of human-biter (autogenous) and bird-biter (anautogenous) forms. These forms are genetically distinct and have been isolated in northern Europe, where they segregate by habitat. The bird-biting mosquitoes live and breed in open areas above-ground and diapause during the winter; human-biting mosquitoes live exclusively in enclosed areas, like the underground rail lines, that are kept warm year-round. Underground human-biting mosquitoes were found to be more closely related to other human-biting mosquitoes from North Africa, the Middle East, Japan, and Australia than to neighboring above-ground specimens. Combined with susceptible migrating birds and highly concentrated human populations, the finding of hybrids in the United States led the authors to hypothesize that hybrid *Cx. pipiens* that may bite both humans and birds have contributed to the unprecedented number of human cases of WNV in North America.

### Tuberculosis

#### Stable Association between Strains of *Mycobacterium tuberculosis* and their Human Host Populations

*Mycobacterium tuberculosis* is a global pathogen that kills two million persons each year. Hirsh et al. investigated whether it is really one and the same *M. tuberculosis* that infects people born in different parts of the world. The evolutionary relationships among 100 *M. tuberculosis* isolates from San Francisco were deduced from the unique genomic sequences deleted from each isolate. For the same 100 isolates, a long-term epidemiologic dataset showed where each isolate's host had been born, and whether he or she had contracted the infection before or after coming to San Francisco. Together, the evolutionary and epidemiologic data showed that a host's place of birth was highly predictive of the genetic identity of the *M. tuberculosis* he or she carried. This pattern held true even among hosts who had been infected after arriving in San Francisco. An estimate of the time separating the genetically divergent groups of *M. tuberculosis* that are carried by persons born in different locations suggested that the associations between human populations and their genetically distinctive strains of *M. tuberculosis* have persisted for centuries.

### About Segue

Segue presents brief summaries of articles on pertinent emerging issues published elsewhere and is edited by Joseph E. McDade.
